# Acute Pericarditis as a Complication of Hiatal Hernia Perforation

**DOI:** 10.7759/cureus.67551

**Published:** 2024-08-22

**Authors:** Piotr Branny, Radim Spacek, David Vician, Alica Cesnakova Konecna, Matej Pekař

**Affiliations:** 1 Cardiothoracic Surgery, Hospital AGEL Třinec-Podlesí, Trinec, CZE; 2 Medicine, Faculty of Medicine, Palacky University, Olomouc, CZE; 3 Cardiology, Hospital AGEL Třinec-Podlesí, Trinec, CZE; 4 Medicine, Third Faculty of Medicine, Charles University, Prague, CZE; 5 Medicine, Faculty of Medicine, University of Ostrava, Ostrava, CZE; 6 Vascular Surgery, Hospital AGEL Třinec-Podlesí, Trinec, CZE; 7 Physiology, Faculty of Medicine, Masaryk University, Brno, CZE

**Keywords:** fistula, mods, septical, stercoral, transverse colon, hiatal hernia, pericarditis

## Abstract

Acute pericarditis is a serious and potentially fatal disease in which a diagnostic workup is not always straightforward. Hiatal hernia, on the other hand, is often asymptomatic and can be easily diagnosed if symptomatic. In advanced forms of hiatal hernia, oppression of intrathoracic organs and heart failure can occur. In uncommon cases, the large intestine can also be translocated into the chest cavity, and very rarely, it can be perforated with the development of mediastinitis and/or pericarditis. We report the case of a 74-year-old female with a 1.5-month history of chest pain with elevated inflammatory markers. This patient was empirically treated with antibiotics for suspected pneumonia. After a few weeks, due to a worsening of the patient's condition, an echocardiogram and then a CT of the chest were performed, showing a large hiatal hernia and a very probable purulent pericarditis, necessitating a surgical exploration. A cardiac surgeon found stercoral contents in the pericardium, with a fistula at the apex of the heart. The operation continued with an exploration of the abdominal cavity; the general surgeon returned the massive hiatal hernia to the abdomen, the contents of which were the stomach and transverse colon. An extensive perforation in the transverse colon was found. Lavage, drainage, and resection of the affected part of the intestine were performed, and a permanent (terminal) colostomy was constructed. The patient was in severe septic shock with multiorgan failure and died 10 hours after surgery despite maximal therapy. This case highlights the importance of interdisciplinary cooperation and the importance of considering the possible fistula in the co-occurrence of hiatal hernia and pericarditis.

## Introduction

Acute pericarditis is the most common disease of the pericardium [1]. The pericardium is a protective, fluid-filled sac that surrounds the heart and helps it function properly. The etiology of pericarditis can be infectious (e.g., viral and bacterial) or non-infectious (e.g., traumatic, systemic inflammatory disease, tumorous); however, the viral etiology is the most common. Diagnosis is based on the presence of clinical symptoms such as specific chest pain, fever, feeling sick, shortness of breath, or paraclinical abnormalities viewed using electrocardiography, echocardiography, or magnetic resonance imaging. The course of the disease is usually mild. However, purulent pericarditis specifically has a high mortality rate. Purulent pericarditis is defined as an infection in the pericardial space that produces macroscopically or microscopically purulent fluid. The treatment is mostly medical, nonsteroidal anti-inflammatory drugs or corticosteroids in the case of idiopathic or viral pericarditis. In bacterial, often purulent pericarditis, antibiotics and pericardial drainage are indicated. If pericarditis is not adequately treated, an inflamed pericardium becomes fibrotic and non-compliant, leading to constrictive pericarditis and significantly reducing cardiac output. Early surgical pericardiectomy with complete decortication is indicated in such cases [2].

Hiatal hernia is a condition in which the abdominal organs transpose to the chest cavity through the esophageal hiatus, which is situated in the muscular part of the diaphragm and transmits the esophagus, the vagus nerves, and some small esophageal arteries. The most frequently dislocated organ is the stomach. However, other intraabdominal organs could be displaced as well. There are four types of hiatal hernias. Type I hernia is referred to as "sliding," in which the lower esophagus and stomach, including the gastroesophageal junction (GEJ), ascend through the hiatus. Type II is called "paraesophageal," where the fundus migrates into the thoracic cavity parallel to the esophagus, but the GEJ remains in the abdominal cavity. In type III, both the GEJ and part of the stomach migrate into the thoracic cavity. Type IV represents the migration of the stomach and other parts of the gastrointestinal tract (intestine, spleen, etc.) into the chest cavity. Asymptomatic hernias do not require specific treatment, and patients undergo regular checkups. Symptomatic ones require surgical repair: reduction and excision of the hernia sac, tension-free repair, recognition and correction of a short esophagus, crural closure with mesh reinforcement, and an anti-reflux procedure [3].

There are published cases of heart failure due to a giant hiatal hernia [4], but pericarditis caused by the perforation of the transverse colon is an extremely rare condition. We performed a PubMed search with the keywords “pericarditis” and “transverse colon” and found only one case published with the pericarditis caused by a fistula from the transverse colon in a patient with ulcerative colitis shown at autopsy [5].

Herein, we present a very rare case of a woman suffering from stercoral pericarditis due to a hiatal hernia with bowel perforation. To the best of our knowledge, this is the first case report where the fistula was found in a living patient.

## Case presentation

**Figure 1:**
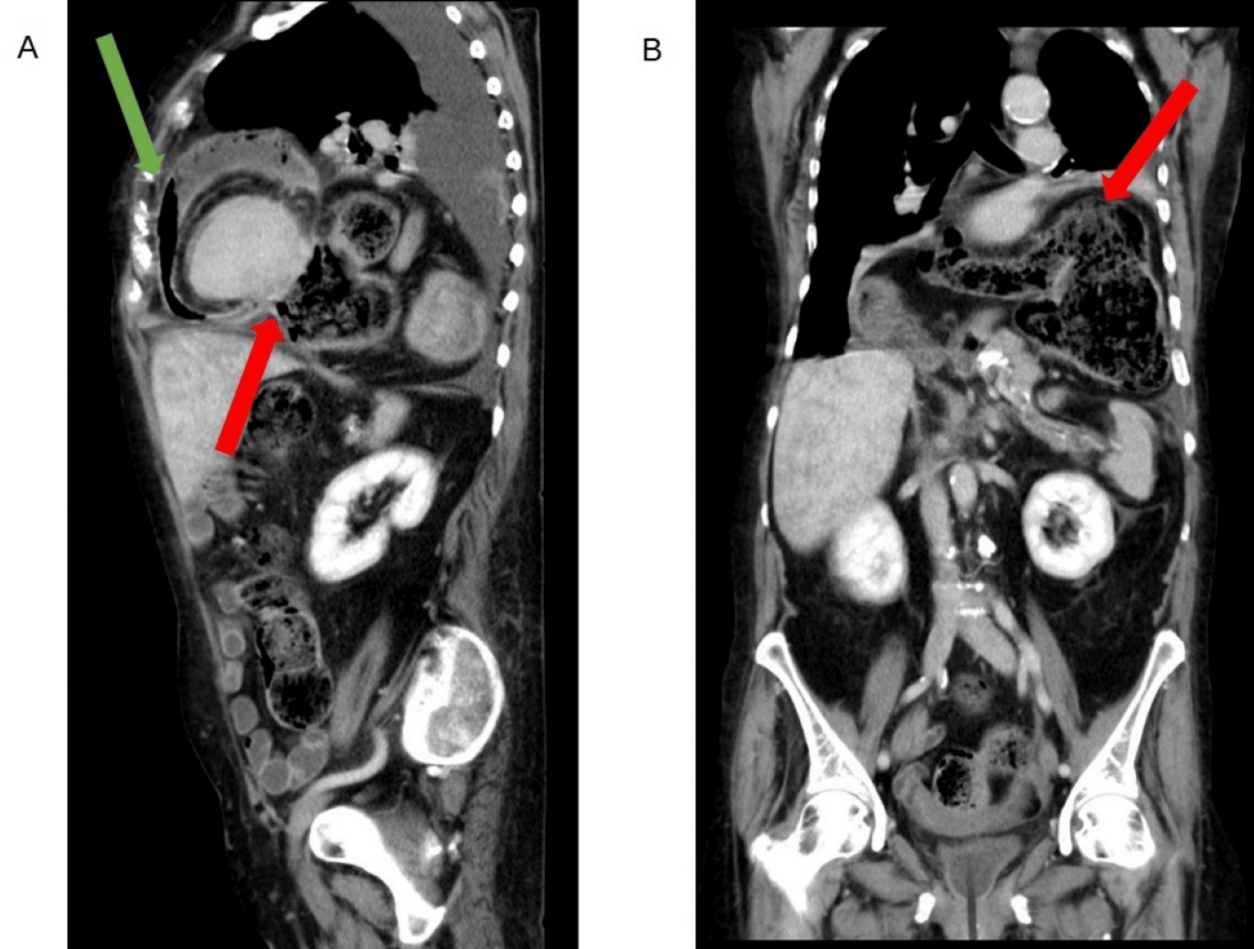
Chest and abdominal CT, venous phase, lateral and anteroposterior reconstruction (A) Lateral reconstruction: the green arrow shows the pericardial effusion with gas, and the red arrow shows the transverse colon perforation with the fistula to the pericardium; (B) anteroposterior reconstruction: the red arrow shows the giant hiatal hernia that compromised the left lung and mediastinum.

**Figure 2:**
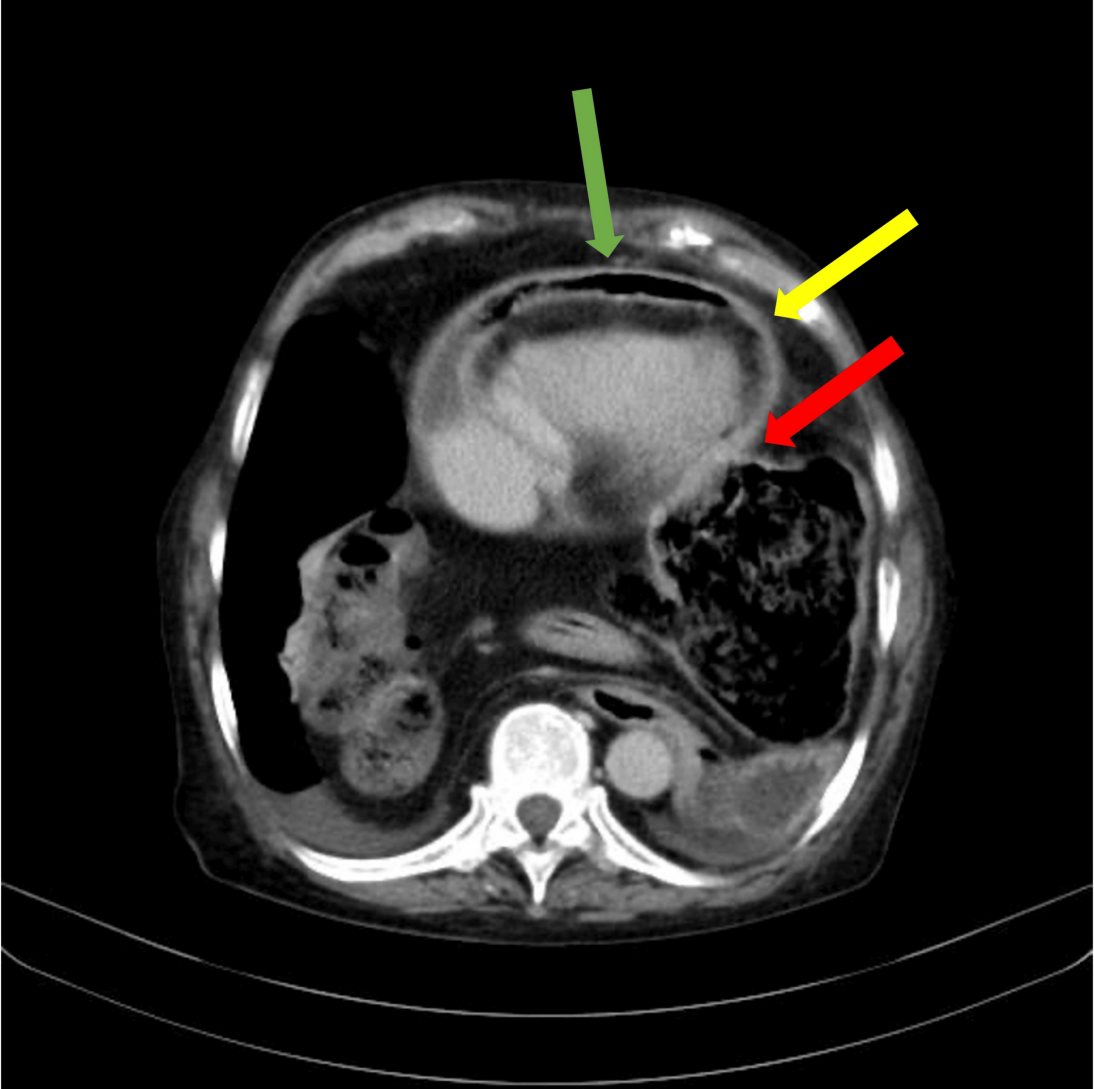
Chest CT, venous phase, 10th thoracic vertebra level, transversal plane The green arrow shows the pericardial effusion with gas. The thickened pericardium (yellow arrow) and the communication of the pericardium with the bowel (red arrow) are also visible.

A 74-year-old woman with a history of arterial hypertension, asthma, mild chronic obstructive pulmonary disease (without chest X-ray findings), and rheumatoid arthritis treated with steroids was transferred from the district hospital to a tertiary cardiology center for purulent pericarditis. The patient's complaints started 1.5 months prior when she was hospitalized for chest pain, dyspnea, and fever. She was treated at a local hospital for suspected pneumonia with antibiotics (ATB) and discharged after a few days. Her condition transiently improved. At home, the patient's dyspnea and loss of appetite persisted, forcing her to be hospitalized again after about three weeks. At admission, she was hemodynamically stable but clinically obviously unwell. Her lab tests revealed a high C-reactive protein (CRP) and a normal leukocyte count. Since the diagnosis was not clear, a computed tomography (CT) scan of the abdomen and chest was performed, where the finding of a hiatal hernia type IV and a suspected purulent pericarditis dominated (Figures 1, 2). The diagnosis of purulent pericarditis was made based on the CT findings, particularly the presence of effusion and gas in the pericardial sac.

**Figure 3:**
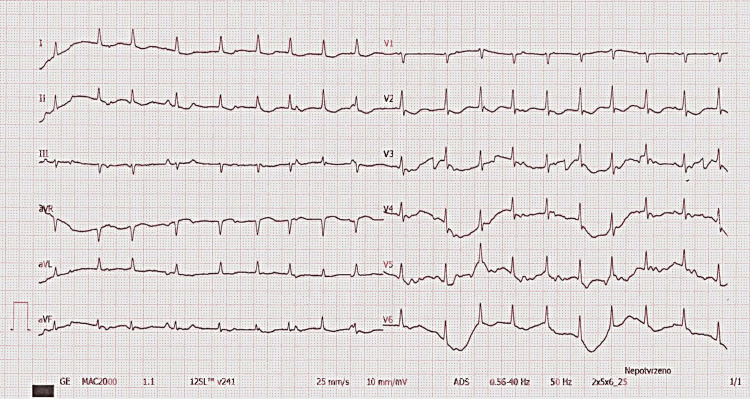
Electrocardiography (ECG) upon admission An ECG recorded upon admission to our center showed multifocal atrial tachycardia. Typical signs of acute pericarditis (PR depressions or ST elevations) are absent.

**Figure 4:**
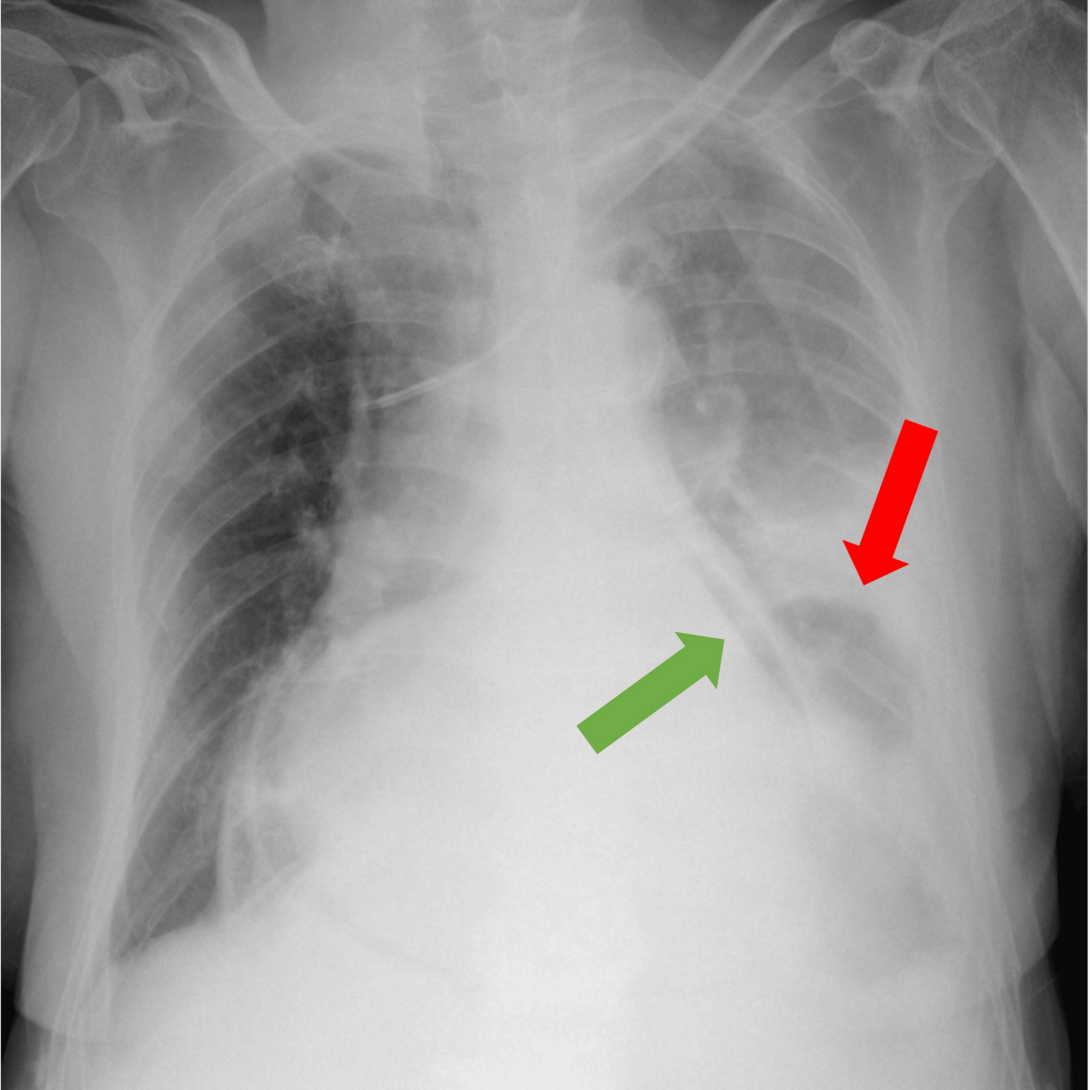
Chest native X-ray in anteroposterior projection The green arrow shows gas in the pericardial area caused by the fistula with gastroenteric communication. The red arrow shows the gastric gas in the chest cavity caused by the giant hiatal hernia.

A transfer to our tertiary cardiology center was arranged to treat the pericarditis. She had a pericardial tap, and echocardiography showed left chamber systolic dysfunction with a 45% ejection fraction and small atypical pericardial effusion (Video 1). Electrocardiography showed multifocal atrial tachycardia (Figure 3). A chest native X-ray showed gas in the pericardial area and gastric gas in the chest cavity (Figure 4). Lab tests revealed a high CRP (Table 1).

**Video 1:** Echocardiography, subxiphoid view, showing atypical small pericardial effusion

**Table 1:** Laboratory tests The table depicts the most relevant laboratory tests during the entire six-day hospitalization period. It shows a decrease in C-reactive protein, an increase in procalcitonin, and significant elevations of procalcitonin and presepsin at the end of hospitalization. CRP: C-reactive protein; ALT: alanine aminotransferase; AST: aspartate transaminase; NT pro-BNP: N-terminal pro-B-type natriuretic peptide.

Day of Hospitalization	1	2	3	4	5	6	Physiological Range
CRP (mg/l)	226	276	285	222	165	53	0-5
Procalcitonin (μg/l)	0.323	-	-	-	-	0.538	0-0.5
Presepsin (ng/l)	-	-	-	-	-	443	0-327
Interleukin 6 (ng/l)	-	-	-	-	-	2172	0-7
Glycemia (μmol/l)	7.7	6.9	9.0	7.1	9.6	8.1	3.5-5.6
Bilirubin (μmol/l)	9.2	-	-	-	7.6	9.9	0-21
Lactate (mmol/l)	-	-	-	-	-	3.08	0.5-2.2
Urea (mmol/l)	8.0	8.7	12.6	14.1	15.1	12.1	2.9-8.2
Creatinin (μmol/l)	54	62	68	81	68	50	45-84
ALT (μkat/l)	0.13	-	-	1.2	3.46	3.28	0.17-0.58
AST (μkat/l)	0.12	-	-	4.23	9.73	5.11	0.17-0.6
Troponin T (ng/l)	-	-	-	-	-	34	0-14
NT pro-BNP (ng/l)	-	-	-	-	-	1687	0-125
Hemoglobin (g/l)	92	92	79	88	93	85	120-160
Leukocyte count (10^9^/l)	8.6	8.6	11.7	15.1	10.7	9.1	4-10

**Figure 5:**
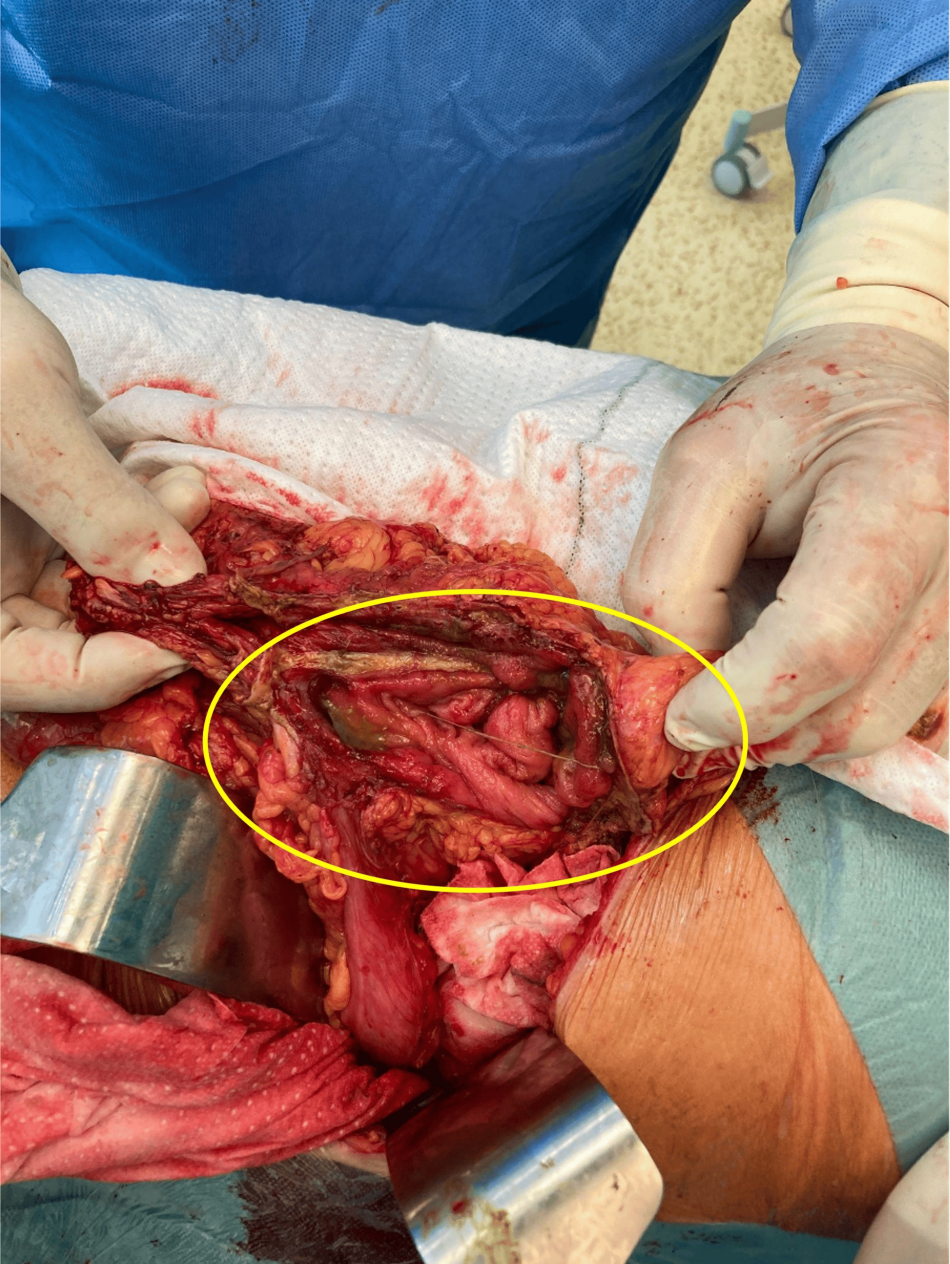
Perioperative finding from the abdominal cavity revision The image depicts the reposited transverse colon from the chest cavity through the esophageal hiatus. The circle shows the bowel perforation with stercoral containment.

At the time of transfer, she started to be hemodynamically unstable, needing vasopressor therapy at the intensive care unit. Blood cultures came back positive for *Staphylococcus hominis*, and she had *Klebsiella pneumoniae* extended-spectrum beta-lactamases (ESBL) in the urine. The ATB therapy was thus escalated (meropenem, vancomycin), and a temporary short-term improvement of the general condition was observed. However, the patient experienced a recurrence of hemodynamic instability, and echocardiography showed signs of constrictive physiology without any pericardial effusion, urgently indicated for cardiac surgery (Video 2). A sternotomy was conducted during the surgery, revealing a thick pericardium that was not connected to the heart. There was a small amount of pus in the pericardial sac but nothing significant. The epicardium exhibited thickening, which likely contributed to the constrictive signs observed on echocardiography. On further exploration after lifting the apex of the heart, we found a fistula with stercoral content. After lavage and drainage of the chest, a general surgeon continued with the exploration of the abdominal cavity. Intraoperatively, the abdominal cavity was clean, without peritonitis, with the finding of a hiatal hernia, which contained the entire stomach and a substantial part of the transverse colon. After repositioning the content of the hiatal hernia back into the abdominal cavity, a large perforation was found on the transverse colon (Figure 5).

**Video 2:** Echocardiography, four-chamber apical view Four-chamber apical view echocardiography shows a typical breathing-dependent ventricular septum movement caused by interventricular dependence, the most specific sign of constrictive physiology.

A 50-cm-long colon resection was performed, followed by the stitching of a terminal transversostomy and blind sealing of the stump. Postoperative procedures included lavage, drainage, and hiatoplasty. The hiatoplasty was constructed respecting the crura anatomy; the stitches of the hiatoplasty were passed from the left to the right diaphragmatic pillar, and the stomach fundus was fixed to the edge of the sutured hiatoplasty. Postoperatively, the patient was admitted to the intensive care unit in critical condition, developing multiple organ dysfunction syndrome (MODS). Despite the complex therapy, including ventilation, vasopressors, antibiotics, and inotropes (levosimendan), it was not possible to ensure adequate hemodynamics. MODS progressed, there was continued anuria, and the condition gradually led to irreversible circulatory arrest. The patient died on the sixth day of hospitalization, 10 hours after the completed surgical revision.

## Discussion

Acute pericarditis is a serious disease that can be fatal. If not treated properly, it can lead to a constrictive form, necessitating pericardectomy. Diagnosis is mainly based on imaging methods, with electrocardiography, echocardiography, and magnetic resonance imaging being the most valuable. Heart catheterization (both right and left) is sometimes needed to definitively reach the diagnosis. The treatment is complex, and up to 30% of the disease recurs [6,7]. A hiatal hernia, on the other hand, is, in the vast majority of cases, a benign condition that does not require direct intervention. If it becomes symptomatic, then surgery to eliminate gastroesophageal reflux and hiatoplasty is indicated [8]. The etiology of hiatal hernias is not completely understood, but quite often, it is a post-inflammatory shortening of the esophagus, leading to the stomach being pulled into the chest cavity [9]. It is very unlikely that a part of the large intestine, which has a common hinge with the stomach, is transposed into the chest cavity along with the stomach. As we found out when searching on PubMed, such a condition is infrequent, and perforation with a fistula into the pericardium in living patients is unseen. The resulting stercoral pericarditis is a serious condition that threatens the patient with sepsis, shock, and multi-organ failure.

In this case report, the patient became hemodynamically unstable, and echocardiography showed signs of constrictive physiology without pericardial effusion, urgently indicating cardiac surgery. We can hypothesize that the effusion emptied into the fistula and uncovered the constrictive physiology. The time of the colon's penetration into the pericardium was not exactly clear, but it must have taken a few weeks because of the large communication and the fact that they were quite firmly attached to each other. The patient has had symptoms for approximately six weeks. ATB therapy certainly slowed down the process of stercoral pericarditis, as antibacterial therapy is mandatory in purulent pericarditis [10]. However, ATB alone was not effective enough to cure the patient. A chest CT scan was the crucial diagnostic modality, showing clear signs of purulent pericarditis with pneumopericardium. A hiatal hernia was a secondary finding, but a fistula was not detected. This condition has not yet been described on CT. The fact that the examination was not with intestinal but only intravenous contrast also contributed to this. The finding of stool in a pericardial cavity during cardiothoracic surgical revision was unexpected. On the other hand, if the fistula was recognized before the surgery, the treatment would be exactly the same. The critical point of this treatment is the timing of the CT scan and the probability of the correct diagnosis, which is very uncommon. Common causes of intestinal perforation include trauma, instrumentation, inflammation, infection, malignancy, ischemia, and obstruction. Our presented patient had no signs of ischemia after bowel repositioning; we can hypothesize that repeating heartbeat could cause the trauma leading to perforation, as well as the chronic steroids therapy. The patient developed a very severe shock with multi-organ failure, with stercoral pericarditis. Thus, it was no longer possible to maintain hemodynamically sufficient circulation in a person with serious comorbidities and at an advanced age.

We found only one similar case with the fistula between the heart and transverse colon, which was reported in 1983 by Greenwood et al. [5]. This patient had open heart surgery with a resection of a left ventricular apical aneurysm. The procedure was uneventful, and there were no postoperative complications until pericarditis developed four years later. Similar to our patient, the patient had a fever, chest pain, anorexia, pericardial tap, and no electrocardiographic changes. On the other hand, there was an absence of a hiatal hernia, and the abscess was found in the anterior pericardium with a fistula through the diaphragm to the left subdiaphragmatic space, where the transverse colon was adjacent to. As it was reported in 1983, there was no CT available, and this condition was only found after the autopsy.

To date, there have been no reported cases of stercoral pericarditis resulting from a fistula originating from the transverse colon in a living patient. It is crucial to consider this diagnosis in case of a concurrent occurrence of pericarditis and hiatal hernia, as it is a highly severe condition that demands immediate medical intervention.

## Conclusions

This case presentation points to the need for interdisciplinary cooperation in the diagnosis and treatment of these patients. It is important to correlate the imaging methods with clinical findings and anamnestic data. In cases of pericarditis and hiatal hernia, with gas in the pericardium, we must consider the possibility of a gastrointestinal fistula with the stercoral pericarditis, which may have fatal consequences.
